# Designing Awe in Virtual Reality: An Experimental Study

**DOI:** 10.3389/fpsyg.2017.02351

**Published:** 2018-01-22

**Authors:** Alice Chirico, Francesco Ferrise, Lorenzo Cordella, Andrea Gaggioli

**Affiliations:** ^1^Department of Psychology, Università Cattolica del Sacro Cuore, Milan, Italy; ^2^Department of Mechanical Engineering, Politecnico di Milano, Milan, Italy; ^3^School of Design, Politecnico di Milano, Milan, Italy; ^4^Applied Technology for Neuro-Psychology Lab, IRCCS Istituto Auxologico Italiano, Milan, Italy

**Keywords:** awe, virtual reality, presence, emotions, emotion induction

## Abstract

Awe is a little-studied emotion with a great transformative potential. Therefore, the interest toward the study of awe’s underlying mechanisms has been increased. Specifically, researchers have been interested in how to reproduce intense feelings of awe within laboratory conditions. It has been proposed that the use of virtual reality (VR) could be an effective way to induce awe in controlled experimental settings, thanks to its ability of providing participants with a sense of “presence,” that is, the subjective feeling of being displaced in another physical or imaginary place. However, the potential of VR as awe-inducing medium has not been fully tested yet. In the present study, we provided an evidence-based design and a validation of four immersive virtual environments (VEs) involving 36 participants in a within-subject design. Of these, three VEs were designed to induce awe, whereas the fourth VE was targeted as an emotionally neutral stimulus. Participants self-reported the extent to which they felt awe, general affect and sense of presence related to each environment. As expected, results showed that awe-VEs could induce significantly higher levels of awe and presence as compared to the neutral VE. Furthermore, these VEs induced significantly more positive than negative affect. These findings supported the potential of immersive VR for inducing awe and provide useful indications for the design of awe-inspiring virtual environments.

## Introduction

Awe is a complex emotion characterized by intense feelings of astonishment, wonder and connectedness that arises when one is confronted with something vast that transcends previous knowledge schemas (Keltner and Haidt, 2003). This emotion can be triggered by natural phenomena, such as the view of the ocean or a starry night, but it may also be elicited by more conceptual contents, such as great works of art and “grand theories” (i.e., the theory of relativity).

In the last decade, the scientific interest of awe has significantly increased, especially because research on positive emotions has shown that experiencing awe is associated with transformative changes at both psychological and physical levels (e.g., Shiota et al., 2007; Schneider, 2009; Stellar et al., 2015). For example, awe can change our perspective toward even unknown others thus increasing our generous attitude toward them (Piff et al., 2015; Prade and Saroglou, 2016) and reducing aggressive behaviors (Yang et al., 2016). Generally, awe broadens our attentional focus (Sung and Yih, 2015), and extends time perception (Rudd et al., 2012). Furthermore, this emotion protects our immunity system against chronic and cardiovascular diseases and enhance our satisfaction toward life (Krause and Hayward, 2015; Stellar et al., 2015).

Considering the transformative potential of awe, an important methodological question concerns how to elicit intense feelings of awe within controlled laboratory conditions (Chirico et al., 2016). However, the intrinsic complexity of this emotion is hard to be reproduced in the lab (Silvia et al., 2015). According to the prototypical model of Keltner and Haidt (2003), awe can be conceived as a complex emotion encompassing the two main appraisal themes of *vastness* (i.e., the perception of being in front of either perceptually or conceptually grand and vast stimuli) and *need for accommodation* (i.e., the urge to adjust mental frames according to new incoming information). Consistently with this operationalization, previous experimental studies on awe have attempted to induce this emotion by exposing participants to grand and vast panorama or natural phenomena (Saroglou et al., 2008; Griskevicius et al., 2010; Van Cappellen and Saroglou, 2012; Van Cappellen et al., 2013) ranging from deep space images, earth and stars (Silvia et al., 2015) to supercell (Gordon et al., 2016). Awe arises from “*information-rich stimuli*,” which entail a need to update our current knowledge of ourselves and the world (Shiota et al., 2007; p. 946).

Recently, virtual reality (VR) has been proposed as a new technique to induce awe in the lab (Chirico et al., 2016). VR is a technology that combines multi-sensory stimuli to generate the perception of being “present” within computer-generated environments and users with the possibility to interact with 3-D contents, resembling real-life interactions, even in a controlled laboratory setting. However, the potential of VR for inducing awe in the lab has not been fully explored yet. A first study carried out by Chirico et al. (2017) showed that the use of immersive videos (i.e., 360-degree video recordings that capture the entire scene around the camera) could generate more intense feelings of awe than conventional 2D videos. Here, we aimed at assessing the effectiveness of computer-generated, three-dimensional virtual environments (VEs) in inducing awe. Differently from immersive videos, VEs are fully interactive graphical scenarios that the user can explore as a ‘real’ physical space. These VEs can be populated with any type of objects and animated characters (i.e. humans, animals, or other creatures) the users can interact with. From a methodological viewpoint, the main advantage is that VEs allow researchers to design any possible scenario and situation, both realistic or imaginary. Furthermore, a VE can be programmed to include specific tasks and challenges, thus widening the range of experimental manipulations. Another possible asset offered by VEs in awe research is that they allow the experimenter accurately tracking participant’s behaviors within the virtual world, i.e., by logging his/her ‘virtual’ actions. Last, but not least, in contrast with 2D videos, a VE can also simulate physically “impossible” phenomena, that is, events breaking the physical or logical laws (i.e., flying over a city, reversing the direction of time etc. (Rosenberg et al., 2013; Friedman et al., 2014; Serino et al., 2015). Finally, immersive VEs are known to generate strong feelings of “presence” in the user, that is, the subjective feeling of being in another physical or imaginary place (Waterworth et al., 2010, 2015; Riva et al., 2011; Riva and Waterworth, 2014; Waterworth and Riva, 2014). Actually, previous research (Diemer et al., 2015) has shown that presence and emotions are closely linked psychological phenomena. Specifically, the feeling of presence can be enhanced by a sense of *immersion* within the VEs (Coelho et al., 2006; Diemer et al., 2015). Immersion consists in the possibility to experience a VE from a first-person perspective and in the sensorial isolation from the real world, as well as in the technological degree of sophistication (Diemer et al., 2015). Crucially, also the possibility to interact with 3-D objects, as a property of immersion, is a key element able to enhance the sense of presence (Blackmon and Mellers, 1996). This VR feature is called *interactivity*. Interactivity has been defined as “the degree of which users of a medium can influence the form or content of the mediated environments” (Steuer, 1992; p. 80). Previous studies have shown that these components of presence (i.e., immersion and interactivity) can amplify emotions reported by users navigating VEs. For example, Baños et al. (2004a) evidence a key relationship between immersive emotional environments and the sense of presence. Specifically, it has been evidenced that immersion can enhance emotional intensity, especially for high arousing emotions (IJsselsteijn et al., 2001; Baños et al., 2008; Diemer et al., 2015). In line with this, Riva et al. (2007) developed a neutral, relaxing and anxious virtual park to test the ability of VR to induce emotions and the link between affects and the sense of presence. They evidenced that the more activating the emotional state was, the higher the sense of presence. However, they focused only on general affective states and not on specific discrete emotions.

On the other hand, Felnhofer et al. (2015) combined visual and auditory cues to design *ad hoc* VR scenarios able to convey specific discrete emotional states (joy, sadness, boredom, anger, and anxiety). They found that the sense of presence was similar across all emotional scenarios, suggesting that presence is not connected to the type of emotion, but maybe, on the presence/absence of an emotional content.

Finally, also realness is a crucial variable conveying a high sense of presence, thus influencing subsequent emotional states (Schubert et al., 2001; Freeman et al., 2005). This would involve several aspects of a VEs, starting from the kind of interaction provided, to the visual and auditory stimuli together (Riva et al., 2007).

Although several studies showed that VR has a potential for both general affect and discrete emotional states, the relationship between complex emotions, such as awe, and VR still needs to be fully explored (Chirico et al., 2017).

In order to test the effectiveness of VE for inducing awe, we designed four different scenarios: three VEs included elements that were assumed to induce different instances of awe (i.e., view of tall trees, high snow mountains, seeing the earth from deep space; please see the Section “Materials and Methods” for a detailed description of VEs). The fourth scenario did not include awe-inspiring elements, and was included as control condition. Our main hypothesis was that awe-VEs would induce greater feelings of awe than the ‘neutral’ VE. Furthermore we expected that presence and awe are positively related, as suggested by previous studies. In other words, the aim of this study was threefold. First, the aim of this research was to validate a set of VR awe-inducing stimuli. Consequently, we were also interested in elucidating which environment was the most awe-inducing one (i.e., the one which induced the higher intensity awe). To this aim, we realized three interactive evidence-based awe-inducing VR environments resembling three instances of awe, following guidelines provided by literature on this complex emotion (Keltner and Haidt, 2003; Shiota et al., 2007; Krause and Hayward, 2015; Piff et al., 2015; Gordon et al., 2016; Yaden et al., 2016). Then, we contrasted the effects of awe, sense of presence and general affect induced by these three environments with those induced by a neutral content created *ad hoc* in VR. Finally, we aimed to advance knowledge in the positive technology field about the efficacy of two different awe-induction techniques: 360°-videos and VR. To this aim, we focused on 360° videos and VR environments to compare their standardized effects on the same variables (i.e., presence and awe). This study has also implications for the methodology of the study of awe. The validated stimuli can be used in other studies to manipulate this emotion.

## Materials and Methods

### Participants

Thirty six participants voluntarily took part in the study (18 females – mean age = 23.33; *SD* = 0.333; 18 males–mean age = 23.67; *SD* = 0.404). Participants were graduate students recruited through campus announcements at an Italian University. We excluded participants reporting vestibular and/or balance disorders. The experimental protocol was approved by the Ethical Committee of the Università Cattolica del Sacro Cuore prior to data collection. Each participant provided written informed consent for study participation. Written consent and all methods were carried out in accordance with the Helsinki Declaration.

### Stimuli

**FIGURE 1:**
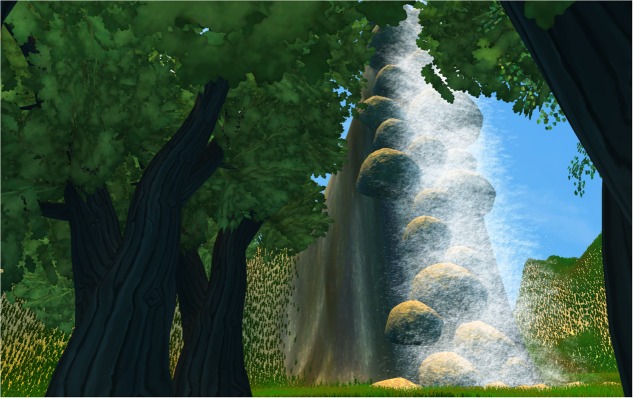
The Forest.

**FIGURE 2:**
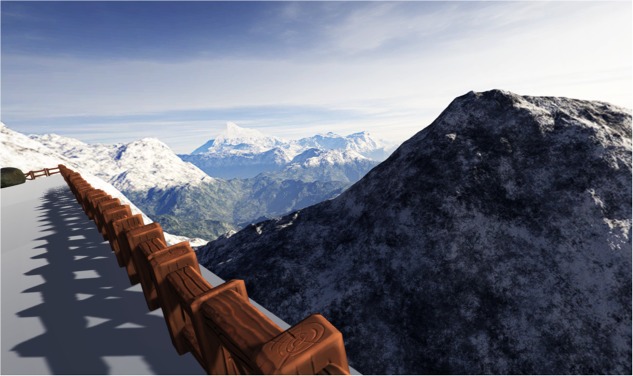
The High Snow Mountains environment.

**FIGURE 3:**
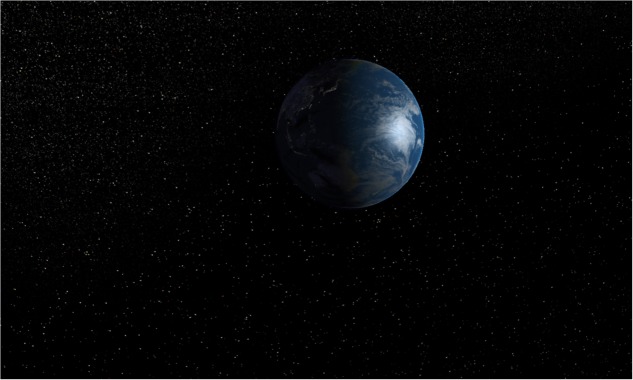
The Earth view from deep space environment.

**FIGURE 4:**
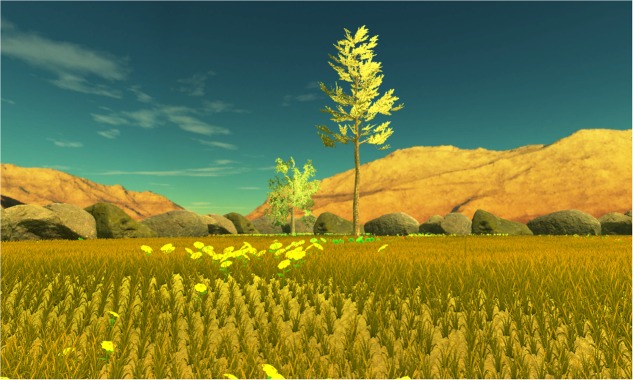
The Neutral environment: green clearing.

We modeled four interactive and immersive VEs with Unity software (version 5.5.1.). Three contents were designed to be awe-inducing, thus they depicted natural scenes of (i) Forest (Piff et al., 2015) (see **Figure 1**); (ii) Mountains (Chirico et al., 2016) (see **Figure 2**) and (iii) Earth view from deep space (Gallagher et al., 2015; Yaden et al., 2016, 2017) (see **Figure 3**). The neutral environments represented a natural scene including green grass with few flowers and trees (see **Figure 4**). To be consistent, we chose only natural scenarios to induce awe, since they are indicated as prototypical awe-eliciting stimuli (Keltner and Haidt, 2003). Further, to enhance the feeling of immersion within the VE, we provided participant with headphones supplied with Oculus rift DK2, so that they could hear the environmental sounds consistently within the virtual landscapes.

Specifically, we followed literature on awe and its sub-components as design principles to be implemented into VR scenarios. First, we drew extensively from the literature on the subdimension of vastness, which emerged as the most crucial component in the experimental study of awe (Chirico et al., 2016).

Since vastness can be either conceptual (in terms of complexity) or perceptual (in physical terms) (Keltner and Haidt, 2003; Yaden et al., 2016), the three target stimuli were designed in line with these two components of vastness. Therefore, this study used an evidenced-based design approach, drawing from literature on awe to “design” vastness. Mountain scenario relied on a concept of vastness as “width” (large panorama), whereas, we designed vastness in terms of “height” (Tall trees and the downfall) in the Forest condition. Finally, the Earth view, which can be conceived as an instance of conceptual vastness (Yaden et al., 2016), was designed to reproduce a prototypical instance of awe, basing on vastness conceptual component.

**FIGURE 5:**
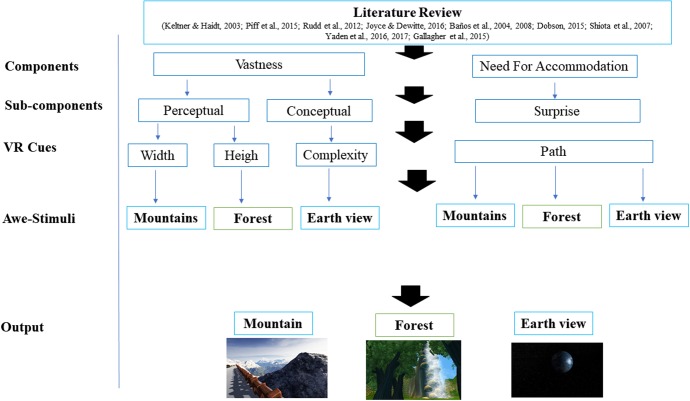
Diagram of evidence-based stimuli design.

Finally, each environment was designed also to induce a need for accommodation as well. Since need for accommodation can be operationalized as a type of surprise (Chirico et al., 2016), we relied on this indication to build the environments, by creating a standardized navigation path leading to an unexpected cue (panorama, waterfall and Tall trees, Earth). See **Figure 5** for the design process of these stimuli.

#### Forest

The composition of this scenario was chosen based on the work of Piff et al. (2015). These Authors led participants to stand in a grove of highest Tasmanian eucalyptus trees. Similarly, our environment displayed a forest of luxuriant forest culminating in a high waterfall, hidden behind trees. Awe is related to high stimuli, both natural (Piff et al., 2015) or artificial (Joye and Dewitte, 2016) and Forest are one of the most prototypical awe elicitors. We chose to enhance the awe-potential of our trees by introducing another highest awe-inspiring stimulus, that is a waterfall (Rudd et al., 2012). Specifically, the waterfall fulfills two important functions. First, the waterfall was used in combination with trees, to enhance the global feeling of vastness. More, since awe is an emotion entailing not only a vastness component (i.e., here the Forest and high waterfall) but also a need for accommodation dimension (i.e., something able to surprise the viewer), we chose to introduce the waterfall behind trees to support this last complex component, which can be assimilated to a special case of surprise (Chirico et al., 2016). Finally, within the forest grove there is a path helping participants to reach the waterfall. We chose to create a standardized path (i.e., the same length and the same navigation speed in all environments) that participants must follow, to prevent them from frustration (Santos et al., 2009). Indeed, we created quite vast virtual space in which participants can navigate but they did not know the environment previously, so, it was possible that they never reached the final target, in this case the waterfall, but just wandering. This would lead to a partial awe experience in which the need for accommodation component would be undermined.

We integrated visual stimulation with consistent environmental sounds including chirping of birds, typical of this kind of forest, along with the noise of wind flowing through the fronds of the trees. Finally, the sound intensity was calibrated according to participants’ proximity to the sound source (i.e., the waterfall falling on the rocks).

#### Mountains

This environment featured high snow mountains with a path of stones culminating in a beautiful panorama. We designed this scenario in line with works of Keltner and Haidt (2003) and Rudd et al. (2012). Here, the vastness component was conveyed by means of the panorama showing the snow-capped picks, while the component of need for accommodation was supported by the unexpected view of the magnificent panorama behind picks. Also in this environment, participants were instructed to follow a path of stones, which led them to the view of panorama, after going through rock walls.

We included the background sound of wind blowing through the peaks. This sound element would enrich users’ experience thus enhancing their sense of presence within the VRE.

#### Earth from Deep Space

Watching at the earth from the space is considered the most prototypical case of awe. This experience was described by White (1987) for the first time as the “overview effect.” Yaden et al. (2016) deepen this phenomenon by analyzing the reports produced by astronauts during space travels. We designed a scenario in which the user is immersed into the deep space in front of the earth. Participants could navigate toward the earth which rotates on itself, as it naturally occurs, showing different sides (illuminated and obscured). In this experience, both vastness and need for accommodation are supposed to be conveyed. Specifically, the vastness is conveyed through the possibility to see something much larger than ourselves and which includes all the people we could have ever known in our lives. The awareness of this condition is related to the newness and to the paradoxical nature of this experience. It is highly unlikely that people could see earth from outside its atmosphere in their lives unless they become astronauts. This contributes to the dimension of the need for accommodation, because it acts as something able to alter people’ accustomed schema, defined as “organized conceptual framework through which individuals approach new information and make sense of old experiences” (Yaden et al., 2016, p. 6).

Here, to be as close as possible to the equivalent real situation, we did not include any kind of sounds, since no sound could be heard in the deep space.

#### Neutral Environment

To induce a non-specific emotive state, we followed guidelines by Baños et al. (2004b, 2008), Riva et al. (2007), Diemer et al. (2015) and we deprived this environment of several other cues used in the other scenarios. Specifically, this scenario displayed a park consisting of a green clearing with very few trees and some flowers. To contrast the effect of vastness conveyed by previous scenarios, we surrounded the whole scene with high stones which hindrance the view outside the woodland. We hypothesized that this physical closure, would be followed by a feeling of psychological closure, the opposite of vastness. Moreover, to ensure that this environment would not convey a need for accommodation, we excluded all elements that could result as unusual or unlikely. For example, the whole environment could be perceived at a glance, nothing was hidden or to be discovered as it happened in the other environments. Participants in this condition followed the same standardized path as in the other conditions (i.e., the same length in all environments).

No sound was included in order not to orient participants’ emotional state.

### Measures

At the baseline, participants were requested to complete:

(1) Again, a single item Likert self-report measure was used to assess *awe* on a 7-point Likert scale (from 1 = not at all; to 7 = extremely), along with other items measuring other seven distinct emotions (from 1 = not at all; to 7 = extremely): Joy, and consensual definitions of emotions taken from literature (Frijda, 1988; Morreall, 1989; Haidt, 2003; Algoe and Haidt, 2009; Griskevicius et al., 2010; Lewis et al., 2010; Herring et al., 2011; Tong, 2015).
(1) *General affect* was assessed by mean of the Italian PANAS version (Terraciano et al., 2003), which measures two main clusters of the affective experience. This questionnaire consists in a list of 20 adjectives measuring the positive affect (10 adjectives) and the negative affect (10 adjectives). This scale showed an adequate reliability for PA (α = 0.76) and for NA (α = 0.83).

After the navigation phase, again, participants’ awe and general affect was assessed. Moreover, also the sense of presence, perceived vastness and need for accommodation were measured:

(1) A single item Likert self-report measure was used to assess *awe* along with other items measuring seven distinct emotions (from 1 = not at all; to 7 = extremely): Anger; Disgust; Fear; Pride; Sadness, Amusement and Joy. This questionnaire was used to obtain a measure of “*global perceived awe.*”
(1) We administered the ITC-Sense of Presence Inventory (ITC-SOPI) (Lessiter et al., 2001) to assess *perceived sense of presence*. It is a well-validated questionnaire composed of 42-items on a 5-point Likert scale (1 = strongly disagree; 5 = Strongly agree). This questionnaire consists in four subscales, each referring to a specific dimension of presence, with a good internal consistency (Cronbach Alpha ranging between 0.76 and 0.94): Sense of Physical Space (0.94); Engagement (0.89); Ecological Validity (0.76); Negative Effects (0.77).
(1) *Perceived vastness* and *Need for accommodation* were assessed through *ad hoc* questionnaire developed by Chirico et al. (2017), according to the guidelines provided by Schurtz et al. (2012) and Piff et al. (2015). Specifically, *Perceived vastness* was assessed using these four items: (1) What I watched provided me with a deep sense of vastness; (2) I felt small in front of what I watched; (3) I felt meaningless in front of what I saw; (4) I felt my sense of self diminish in front of what I saw). *Perceived need* for accommodation was measured through four items: (1) It was hard to grasp what was going on in the video; (2) I felt confused and bewildered in front of what saw; (3) I was struck by the video).

### Procedure

First, participants read and signed the informed consent document. Then, they were given a written and an oral description of the study. They were seated on a chair in front of a computer, and they completed the baseline measures. Then, they were asked to stand up in front of a computer and were tested once per session. Before the navigation phase, participant received a set of instructions about the experiment. The experiment was divided into two phases: baseline and navigation. In the baseline phase, participants were required to report their general affect (PANAS) and the extent to which they felt seven discrete emotions including awe. Then, they were provided with information about how to use the Oculus Rift DK2 and its fitted Microsoft Xbox controller (plugged to the laptop). Finally, they navigated in each VE once in a counterbalanced order. The Oculus Rift DK2 (Oculus VR LLC, Irvine, CA, United States) is a head-mounted display (HMD) with a resolution of 1920 × 1080 pixels and a frame rate of 90 Hz. And also, to avoid cybersickness, we used the following computer: graphic card NVIDIA GTX1070, and a CPU Intel i7. More, we optimized the VEs to avoid low framerates and screen flickering. Immediately before wearing the HMD, participants were instructed to close their eyes until the VE was displayed. The same VE was visible on a desktop computer (i.e., Virtual Desktop application developed for the Oculus Rift), so that also the experimenter could check for it to start correctly. Then, participants received standardized instructions about how to navigate in each environment. The instruction form was the same for all environments, as well as the length of the initial path they could navigate in.

Standardized instructions format was as follows:

“*Please, follow* (specific natural cues embedded into the VEs to provide participants with the same task: stones, a path, the earth), *and then explore the virtual environment freely, as you prefer.*”

When participants indicated that they were ready to begin, the experimenter started countdown. The navigation phase lasted 3 min. After each VE, participants completed the self-reported measures described above. This procedure was repeated four times, one time for each condition, with each participant. After all, four environments had been experienced by the subjects, a debriefing phase concluded the session and interviews were recorded. The entire experiment lasted about 55 min.

## Results

### Data Analyses

Two normality tests (i.e., Kolmogorov–Smirnov and Shapiro–Wilk) were carried out to determine if variables were normally distributed. Only positive affect dimensions, sense of perceived vastness, sense of physical presence, perceived engagement in each condition were normally distributed. Also, ecological validity in the High snow mountains and in the Neutral condition followed a normal distribution. Given the small size of our sample, we chose to carry out parametrical statistical analyses for normally distributed variables (i.e., repeated measures ANOVA) and the equivalent not parametrical tests for not normally distributed variables (i.e., Friedman test and Wilcoxon Signed Rank tests).

### Discriminant Ability of Each Virtual Environment

Results showed that the three awe-inducing VEs induced higher levels of awe compared to the neutral stimulus and to the baseline. **Table 1** shows descriptive statistics concerning emotions scores for each condition.

**Table 1:** Emotions scores for each condition: mean and standard deviation for each emotion in each condition.

Measure	Baseline		Forest		Mountains		Earth view		Neutral	
					
	Mean	*SD*	Mean	*SD*	Mean	*SD*	Mean	*SD*	Mean	*SD*
Anger	1.889	1.214	2.167	1.648	1.472	0.696	1.667	1.352	1.972	1.521
Disgust	1.472	0.941	1.389	1.202	1.333	0.756	1.250	0.770	1.778	1.333
Fear	1.861	1.046	1.472	1.055	2.417	1.713	1.611	1.202	1.361	0.682
Pride	2.778	1.570	2.333	1.219	2.611	1.379	2.361	1.397	2.111	1.214
Amusement	2.500	1.424	2.333	1.493	2.750	1.680	2.417	1.574	2.056	1.012
Sadness	1.861	1.334	1.306	0.822	1.444	0.909	1.278	0.741	1.444	1.027
Joy	2.806	1.582	3.389	1.777	3.694	2.215	3.194	1.940	2.556	1.361
Awe	2.222	1.417	4.556	1.647	5.250	1.697	4.611	1.856	3.194	1.546


### Awe

Awe variable was not normally distributed across conditions; thus, we chose to carry out a Friedman test of differences among repeated measures (all conditions including the baseline) was conducted and rendered a Chi-square value of 73.07 which was significant (*p* < 0.001). Then, a Wilcoxon Signed Rank test to compare awe levels across all environments including the baseline. When multiple statistics are applied to discover pairwise associations, it is necessary to adjust the significance levels since the probability to commit Type I errors increases. With this regard, Bonferroni correction lowers the critical *p*-value for the Wilcoxon test and it relies on the number of performed tests. Therefore, for adjustment, we computed the corrected level of significance (α = 0.05) to address the multiple statistics. Since we had 5 conditions the number of possible combinations is 10 (=5^∗^4/2) [N(N-1)/2] and we adjusted the significance level to 0.005 (=0.05/10) (Cabin and Mitchell, 2000). Resulted showed that Forest [Mdn = 6.00; *Z* = -2.406, *p* < 0.001; *r* = 0.294], Mountains [Mdn = 5.00; *Z* = -4.635, *p* < 0.001; *r* = 0.546], Earth [Mdn = 5.00; *Z* = -3.557, *p* < 0.001; *r* = 0.419] induced significantly higher levels of awe compared to the Neutral stimulus (Mdn = 3.00). Forest [*Z* = -4.852, *p* < 0.001; *r* = 0.571], Mountains [*Z* = -4.984, *p* < 0.001; *r* = 0.576], Earth view [*Z* = -4.969, *p* < 0.001; *r* = 0.585] significantly differed from the baseline (Mdn = 2.00) regarding awe levels. No significant difference emerged between baseline awe and awe induced by the Neutral environment.

Please, see **Table 2** for Wilcoxon test between each condition and the baseline for each emotion.

**Table 2:** Significance levels for each condition compared to baseline using Wilcoxon test.

Measure	Forest vs. baseline		Mountains vs. baseline		Earth view vs. baseline		Neutral vs. baseline	
				
	*Z*	*p*-value	*Z*	*p*-value	*Z*	*p*-value	*Z*	*p*-value
Anger	-0.726	0.468	-1.908	0.056	-0.861	0.389	-0.158	0.874
Disgust	-0.574	0.566	-0.615	0.538	-1.254	0.21	-1.152	0.249
Fear	-1.65	0.099	-1.733	0.083	-0.753	0.451	-2.165	0.03
Pride	-1.418	0.156	-0.853	0.394	-1.722	0.085	-2.392	0.017
Amusement	-0.667	0.505	-0.569	0.569	-0.301	0.764	-1.569	0.117
Sadness	-2.312	0.021	-2.025	0.043	-2.371	0.018	-1.917	0.055
Joy	-1.941	0.052	-2.295	0.022	-0.94	0.347	-0.848	0.396
Awe	-4.852	<0.000^∗^	-4.984	<0.000^∗^	-4.969	<0.000^∗^	-2.335	0.02


*Significance at *p* < 0.001 = ^∗^.*

Finally, we carried out also Wilcoxon Signed Rank test to compare awe levels only across the three target conditions (i.e., Forest, Mountain, Earth), in order to elucidate which environment was the most awe-inducing one. For adjustment, we computed the corrected level of significance (α = 0.05) to address the multiple statistics. Since we had only 3 conditions, the number of possible combinations is 3 (=3^∗^2/2) [N(N-1)/2] and we adjusted the significance level to 0.017.

Results showed that Mountain [Mdn = 6.00; *Z* = -2.406, *p* < 0.01; *r* = 0.232] induced significantly higher levels of awe compared to Forest (Mdn = 5.00) and to Earth (Mdn = 5.00). No significant difference between Forest and Earth was evidenced.

### Sense of Perceived Vastness and Perceived Need for Accommodation

We carried out a one-way repeated measures ANOVA (conditions: Forest, Mountains, Earth view, Neutral), with vastness as a measure. It emerged a statistically significant effect of condition on the sense of perceived vastness [*F*(3) = 29.526; *p* < 0.001; η^2^ = 0.458]. *Post hoc* comparisons were made to determine the significance of pairwise contrasts, using the Bonferroni correction (α = 0.05). High snow mountains elicited a significantly higher sense of perceived vastness (mean =18.527; *SD* = 4.953) compared both to Forest (mean = 15.889; *SD* = 5.306) and the Neutral stimulus (mean = 11.500; *SD* = 4.982). Mountains and Earth view (mean = 18.278; *SD* = 6.738) did not significantly differ regarding the ability to convey a sense of vastness. Earth view conveyed a higher sense of vastness than the Neutral stimulus only. No statistically significant difference emerged regarding perceived need for accommodation across conditions.

**FIGURE 6:**
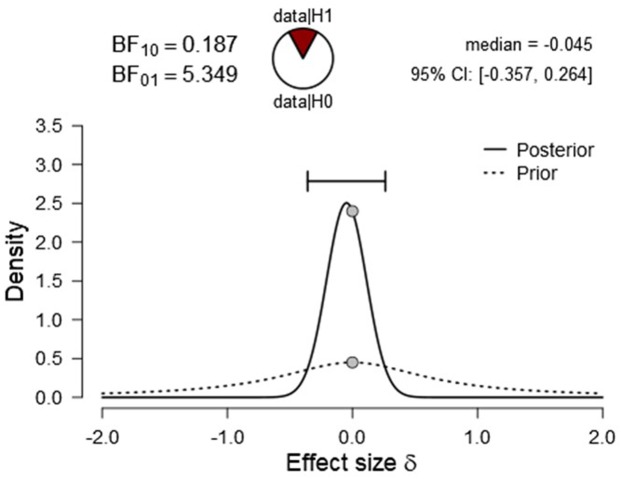
Bayesian graph of Mountains vs. Earth view conditions considering vastness measure.

To test whether Mountains and Earth view were statistically similar in conveying vastness, we carried out the paired sample *t*-test Bayes Factor (BF), namely a ratio between the likelihood of the data given null-hypothesis and the one given the alternative one (Masson, 2011; Liang et al., 2012; Nuzzo, 2014; Rouder, 2014), using JASP (see **Figure 6**).

Results showed that Mountains and Earth view, the most vastness-conductive stimuli, were statistically significant regarding their ability to convey vastness. Results evidenced a substantial effect for the condition (BF_01_ = 5.349; err = 2.093e-8). In other words, Mountains elicited levels of vastness significantly similar to the Earth view condition.

### Presence

First, we carried out a one-way repeated measures ANOVAs (conditions: Forest, Mountains, Earth view, Neutral), with the two normally distributed dimensions of presence as measures (i.e., physical presence and engagement).

#### Physical Presence

There was a statistical significant effect of condition on the perceived physical presence [*F*(3) = 29.180; *p* < 0.001; η^2^ = 0.456]. *Post hoc* comparisons were made to determine the significance of pairwise contrasts, using the Bonferroni correction (α = 0.05). Results showed that Forest, Mountains, significantly enhanced the perceived sense of being physically present within the VE more than the Neutral environment. Contrarily, Earth view induced a significantly lower level of physical presence than the neutral condition. In other words, the Earth view condition induced the lowest level of perceived physical presence compared to other conditions. See **Table 3** for descriptive statistics.

**Table 3:** Physical presence and engagement scores for each condition: Mean and standard deviation for each condition.

Measure	Forest		Mountains		Earth view		Neutral	
				
	Mean	*SD*	Mean	*SD*	Mean	*SD*	Mean	*SD*
Physical Presence	3.402	0.862	3.382	0.825	2.545	0.8706	3.048	0.929
Engagement	3.660	0.824	3.754	0.874	3.20	0.985	3.305	0.914


#### Engagement

There was a statistical significant effect of condition on perceived engagement [*F*(3) = 9.964; *p* < 0.001; η^2^ = 0.222]. *Post hoc* comparisons were made to determine the significance of pairwise contrasts, using the Bonferroni correction (α = 0.05). Again, results showed that Forest and Mountains conveyed significantly higher levels of engagement compared with the Neutral environment. However, Earth view induced significantly lower levels of engagement compared to the other two target conditions. See **Table 3** for descriptive statistics.

#### Ecological Validity and Negative Effect

First, we carried out Friedman test of differences among repeated measures (including the four conditions) was conducted and rendered a Chi-square value of 27.42 which was significant (*p* < 0.001). Secondly, we computed Wilcoxon Signed Rank test adjusting alpha as mentioned in the “awe” results section both with ecological validity and negative effect as measures. Here, we had 4 conditions, so the number of possible combinations was 4 (=4^∗^2/2) [N(N-1)/2], consequently, we adjusted the significance level to 0.008 (=0.05/6). Resulted showed that Mountains (Mdn = 4.00) induced the highest levels of ecological validity compared to Forest [Mdn = 3.60; *Z* = -3.118, *p* < 0.001; *r* = 0.424], Earth view [Mdn = 3.50; *Z* = -4.335, *p* < 0.001; *r* = 0.895] and the Neutral environment [Mdn = 3.60; *Z* = -3.531, *p* < 0.001; *r* = 0.416]. Regarding negative affect, scores ranged between 1 and 2 on a 5 point-likert scale. Friedman test showed a Chi-square 9.26 (*p* < 0.05). We carried out a Wilcoxon Signed Rank test which showed that Mountains (Mdn = 1.500) elicited significantly higher levels of negative affects compared to Earth view condition [Mdn = 1.167; *Z* = -2.702, *p* < 0.001; *r* = 0.602]. Neutral stimulus (Mdn = 1.500) induced higher levels of negative affect compared to Forest [Mdn = 1.500; *Z* = -2.834, *p* < 0.001; *r* = 0.334] and Earth view conditions [*Z* = -2.769, *p* < 0.001; *r* = 0.326]. Consider **Table 4** for descriptive statistics.

**Table 4:** Ecological validity and negative effect scores for each condition: Mean and standard deviation for each condition.

Measure	Forest		Mountains		Earth view		Neutral	
				
	Mean	*SD*	Mean	*SD*	Mean	*SD*	Mean	*SD*
Ecological validity	3.506	0.998	3.937	0.9213	3.178	1.1854	3.389	1.0715
Negative effects	1.718	0.959	1.991	1.034	1.472	0.5697	1.982	1.1054


### Positive vs. Negative Affect

Since, awe is a composite emotion with a not well-defined valence, we chose to integrate the discrete approach to emotions, with a dimensional one, which allows capturing nuances of positive and negative affective states associated with each environment. First, we carried out a separate *t*-tests to investigate the differences of in the positive affect before (baseline) and after the exploration of environments. No significant difference was found for Forest, Earth view and Neutral environment with respect to the baseline. However, exposure to High snow mountains significantly increased positive affect (mean = 3.461; *SD* = 0.714) compared to the baseline condition (mean = 3.161; *SD* = 0.512) [*t*(*35*) = -2.900; *p* < 0.01].

More, we carried out a Friedman test of differences among repeated measures, considering negative affect as a measure (including all the four conditions and the baseline) which rendered a Chi-square value of 33.51 (*p* < 0.001). A separate Wilcoxon Signed Rank tests to investigate the differences in the negative affect before (baseline) and after the exploration of environments. Forest [Mdn = 1.1; *Z* = -4.003,*p* < 0.001; *r* = 0.471], Mountains [Mdn = 1.1;*Z* = -3.092, *p* < 0.001; *r* = 0.364], Earth view [Mdn = 1; *Z* = -4.135, *p* < 0.001; *r* = 0.487] and Neutral condition [Mdn = 1; *Z* = -2.837, *p* < 0.001; *r* = 0.334] significantly decreased negative affect compared to the baseline. Overall, all environments conveyed positive affect and significantly reduced the intensity of negative one. High mountains resulted as the most positive affect conductive, able to significantly increase participants’ positive moods.

Secondly, we carried out a one-way repeated measures ANOVA considering all four conditions (Forest, Mountains, Earth view, Neutral) with positive affect as a measure. There was a statistical significant effect of condition on the perceived general positive affect [*F*(3) = 29.180; *p* < 0.05; η^2^ = 0.08]. *Post hoc* comparisons using the Bonferroni correction showed that only the Neutral condition (mean = 3.116; *SD* = 0.870) elicited significantly lower level of positive affect only compared with the Mountain condition (mean = 3.461; *SD* = 0.714). No significant effect for negative effect across condition was found after correcting with Bonferroni. However, even not significant, High snow mountains displayed the highest level of negative affect (mean = 1.289: *SD* = 0.432) followed, respectively, by the Neutral stimulus (mean = 1.286; *SD* = 0.3766), the Forest (mean 1.228; *SD* = 0.366) and the Earth view (mean = 1.172; *SD* = 0.3141).

### 360° Awe-Inspiring Contents vs. VR Awe-Inspiring Contents

To compare the effectiveness of VR and 360° as two novel emotion-induction techniques, regarding awe induction, we contrasted effect sizes from Chirico et al. (2017) with effect size calculated in this experiment. Specifically, we focused on two measures: awe and sense of presence.

First, since we chose to compare awe scores in this study with those calculated in a previous study (Chirico et al., 2017) with a similar experimental design, we had to be consistent with the distribution of data. In this current study, perceived awe measure was not normally distributed, thus we calculated effect size starting from *z*-score from Wilcoxon Signed Rank test. We computed effect size from the previous study by comparing 360° awe-inspiring video and 360° neutral video regarding the ability to convey a feeling of awe, following the same statistical procedure (we carried out a Wilcoxon Signed Rank test). Results showed an effect size (computed as “r”) of 0.58 [*Z* = -5.367, *p* < 0.001; *r* = 0.471]. On the other hand, we compared these effect sizes with those calculated in the current study. In this study, we computed effect size of awe by comparing each awe-inspiring condition with the neutral VRE. Effect size (r) calculated from the comparison between Forest and Neutral condition was 0.294. Mountains vs. Neutral effect size was *r* = 0.546. Earth view vs. neutral effect size is 0.419. 360° awe-inspiring videos resulted as more effective than VR awe-inspiring environments, although the difference was not wide.

Moreover, Chirico et al. (2017) considered only engagement and physical presence as measures for the sense of presence. Therefore, in the current study, we considered engagement and physical presence as dimensions of presence.

First, we used data from Chirico et al. (2017) to compute a repeated measure ANOVA comparing 360° awe-inspiring video with 360° neutral video with engagement as a measure. Results showed a statistical significant effect of condition on engagement [*F*(1,41) = 48.846; *p* < 0.001; η^2^ = 0.544]. Then, we compared this effect size with those calculated in the current study. Particularly, we calculated effect size (η^2^) comparing each awe-inspiring environment of the current study with the neutral condition. Effect size from Forest vs. Neutral comparison is 0.560. Effect size from Mountains and Neutral is 0.571. Effect size from Earth view vs. Neutral is 0.390. Both virtual Forest and Mountains resulted as more effective in inducing higher levels of engagement compared to 360° videos. Finally, again, we used data from Chirico et al. (2017) to compute a repeated measure ANOVA comparing 360° awe-inspiring video with 360° neutral video with physical presence as a measure. Results showed a statistical significant effect of condition on physical presence [*F*(1,41) = 125.045; *p* < 0.001; η^2^ = 0.753]. Then, we calculated effect size (η^2^) comparing each awe-inspiring environment of the current study with the neutral condition. Effect size from Forest vs. Neutral comparison is 0.236. Effect size from Mountains and Neutral is 0.246. Effect size from Earth view and Neutral is 0.381. Physical presence was higher in the 360° study, considering a 360° neutral environment as a comparison condition.

## Discussion

In this study, we tested the possibility of inducing awe through VR. To this end, four immersive and interactive VEs were developed to induce awe and a neutral emotional state, according to guidelines provided by literature (Piff et al., 2015; Yaden et al., 2016, 2017; Chirico et al., 2016, 2017). As hypothesized, the three target environments (i.e., Tall trees, High snow mountains, Earth view) induced a significantly greater awe than the Neutral stimulus (i.e., cleaning). Furthermore, each stimulus induced more awe compared to other potentially intervenient emotions (Chirico et al., 2017). In other words, it emerged the possibility to elicit awe even using interactive elicitors. This is far more relevant if we consider that awe is considered as a contemplative emotion (Darbor et al., 2016).

Further, our aim was also to elucidate which environment was the most awe-inducing one (i.e., the one which induced the higher intensity awe). This study demonstrated that Mountain environment was the most awe-conductive one. Specifically, High snow mountains scenario displayed slightly higher level of fear and joy, as well as of vastness and need for accommodation if compared to the other two target stimuli. With this regard, Keltner and Haidt (2003) stated that awe could be elicited by stimuli perceived as conceptually and perceptually vast. A recent article by Yaden et al. (2016) identified the overview effect as a case of conceptual vastness “Vastness can be (..) conceptual (..) as (..) the fragility and complexity of life on a small planet in the vastness of space” (p. 4). On the other hand, Mountain and Forest are usually conceived as perceptual instances of awe (Yaden et al., 2016). This is the first study that focus on the ability of conveying awe by two perceptual and conceptual instances of this emotion. Specifically, this research evidenced that High snow mountains stimulus, which was designed to convey vastness through a large panorama, was the most effective elicitor of awe. Surprisingly, also Earth scenario, which can be conceived as a conceptual instance of awe, was able to convey a sense of vastness statistically significant to that induced by High snow mountains. This result can be extended also to the need for accommodation component. That is, all three target scenarios did not significantly differ regarding their ability to convey need for accommodation. This suggests that High snow mountains and Earth view, which can be conceptualized as perceptual and conceptual instances of awe, were equivalent regarding their ability to convey both a sense of vastness and a need for accommodation. However, High snow mountains and Earth view did not convey more need for accommodation than Forest stimulus. Far from being an outcome of vastness and need for accommodation components manipulation, these results can suggest how it is possible to design interactive virtual scenarios able to target different facets of awe. Future studies can take inspiration from these findings to address a more controlled manipulation of such awe components.

For instance, this study evidenced that empirical translation of vastness in terms of “width” worked better in VR compared to that in terms of “height,” and that different instances of vastness can be equivalent into a VR setting.

Moreover, this study is in line with findings from Reinerman-Jones et al. (2013), Gallagher et al. (2014, 2015), Quesnel and Riecke (2017) who demonstrated that VR was able to generate awe through one of the most prototypical experiences of this emotion, that is, the overview effect (i.e., the experience of viewing landscapes from far above, which includes the Earth view as the “quintessential version of this experience”; Yaden et al., 2016, p.2). Here, we showed that the overview effect, when displayed in VR, can be considered closer to natural-based VR experiences. One of the main features of the overview effect it is its intrinsically extraordinary nature. It is very alike that we can see earth from outside its atmosphere during our life. Therefore, it is the component of the need for accommodation that is stressed in this experience. Indeed, if vastness changed significantly across conditions, the need for accommodation did not. In other words, VR could be considered as a sort of natural generator of need for accommodation. It is noted that VR itself could be considered as a source of paradoxical, unusual experiences (e.g., Friedman et al., 2014; Gaggioli, 2016; Gaggioli et al., 2016; Pallavicini et al., 2016; Serino et al., 2016) this would open to a broad array of possibilities regarding the creation of VR scenarios for eliciting complex emotions in the lab. First, these findings indicated that it is possible to induce vastness and the need accommodation components of awe differentially. This can be promising for the design of VR environments able to induce even other complex awe-related states, such as the emotion of admiration (Onu et al., 2016) or elevation (Haidt, 2000), or to manipulate different components of awe effectively. According to the prototypical model of Keltner and Haidt (2003) on awe, this emotion is structured as a family. In the core part of this family there is the prototypical awe, and it is possible to find other complex emotions around it, which are closely connected to awe, such as surprise, admiration or elevation. These VR scenarios are a promising starting point for the design of other awe-family emotional states, allowing also an integrated assessment of theme (combining physiological, behavioral and self-reported measures).

At a more general level, all these environments conveyed also a high positive affect. Consistently with our purposes, the Neutral environment elicited the lower positive affect. Interestingly, High snow mountains induced a higher level of negative effect compared to all other conditions, but the only relevant difference was between the Earth view and the Neutral stimulus. At the design level, it emerged that the Earth view condition led to less negative effects related to navigation, maybe because there were less reference points that could make participants feel disoriented. This hypothesis is in line with qualitative reports of participants at the end of the experimenter. Indeed, two main themes emerged regarding the use of each environment. First, the circular trajectory of navigation of the Neutral stimulus was perceived as monotonous and disorienting. Secondly, the possibility to see a vast panorama near to a cliff, was perceived as dangerous but extremely fascinating, thus leading to a feeling of disorientation. Therefore, two different kinds of disorientation emerged. The first, which regarded the Neutral stimulus, was related to the navigation trajectory. The second one, which concerned the Mountains, consisted of the expected feeling of disorientation related to intense episodes of awe. However, the relevance of this effect is limited since ecological validity scores ranges from 1.5 to 1.7 on a 7-point Likert scale.

Furthermore, Forest and Mountain resulted as more engaging than the Neutral environment, as well as more presence conductive. Unexpectedly, Earth view environment induced a lower sense of engagement and physical presence, as dimensions of presence, than the Neutral environment. This could be due to the intrinsic paradoxical nature of this experience, which led to a decreasing in the sense of physical presence (i.e., participants struggled to believe and feel as if they were there). On the other hand, a reason for lower engagement levels could be that this experience was almost static (in the deep space perception of movement is different from the one perceive on Earth) and totally silent. This could have led to an unusual perception that contributed to a less engaging experience.

Together all this evidence showed that awe can be elicited at high intensity even in the lab. Specifically, mean scores of awe, in awe-inducing environments, ranged from 4.611 to 5.250 on a 7 point Likert scale and the distribution of awe variable showed a high negative skewness. This showed that the bulk of the values lie to the right of the mean, that is, closer to the maximum score of awe (Kim, 2013). Specifically, compared to the baseline, each target environment elicited a significantly higher amount of awe. More, each target condition elicited also other emotions besides awe, compared to the baseline. At the same time, also the neutral condition resulted as inducing different emotional states (i.e., pride, awe, fear) compared with the baseline. However, the neutrality of an emotional stimulus should be evaluated also in relation to other emotional material (Dan-Glauser and Scherer, 2011; Piff et al., 2015). With this regard, our results supported the idea that our Neutral condition acted as a control condition if compared with the other VEs. These results suggested that VR alone, cannot induce intense awe states, but also an *ad hoc* content is required (Chirico et al., 2017). This result was also supported in this study. Even though the neutral stimulus was able to induce a higher amount of awe compared to the baseline, it was not as high as that induced by other VEs whose content was designed to induce awe.

Finally, also each target condition elicited awe as well as other emotions. This effect is well-known in emotion induction research as it is common that a stimulus induces other emotions beyond the target one. However, it is relevant that other intervenient emotions are not higher (in mean) compared to the target emotion (i.e., awe) (Mayer et al., 1995), even they can be considered in line with awe sub-components (joy, fear).

In other words, results showed a satisfactory degree of specificity in emotion induction for our stimuli, therefore, VR emerged as an effective inductor of awe and its sub-components.

With this regard, even Chirico et al. (2017) tested the potential of immersive videos (highly realistic and immersive videos displaying natural scenes) in inducing awe, they had only scratched the surface. In other words, they have only addressed the first part of the continuum of interactive technologies. They considered the lowest level of interaction with VE (i.e., head-tracking). Here, we moved forward by improving their previous emotion-technique and we tested a more advanced form of awe-inducing technique, as well as more complex forms of interactions. In other words, we compared the effect of 360° videos and VR scenarios developed *ad hoc* for this study on awe and sense of presence. Indeed, there are many differences between these two forms of VR. First, the representational apparatus of 360°-videos is composed of a camera situated in a real time-space environment, while VR adopts a 3-D representational apparatus, which is sensitive to users’ input at many levels. At the same time, these two media conveyed the feeling of “presence” differently. 360°-videos can ensure a lower sense of presence compared to VR in which a user is physically located in the space of the video camera. This was reflected into our comparison between 360°-videos and VR. We carried out a direct comparison between 360° videos and VR based on effect size. VR enhanced the sense of engagement – component of presence – more than 360° videos. However, 360° videos increased awe intensity more than VR. This could be due to the requirement to interact and navigate in an unfamiliar VR environment that we gave to participants. While in the 360°-video participants had a sort of omniscient view of the scene and subsequent higher sense of control, users in the VR settings could have perceived a lack of control on the scene. This could affect awe rates. Indeed, this emotion entails a sense of uncertainty which can be tolerated only at some extent (Valdesolo and Graham, 2014).

However, there was a small difference in awe induced by VR vs. 360°, thus, we suggested VR, compared to 360° videos, to induce an intense awe for several reasons. First, VR environments allow for a more ecological interaction with virtual content closer to the equivalent real one. Here, we proposed a higher form of interaction (i.e., navigation within a VE), but it is possible that future works could focus on more sophisticated ones. For instance, it could be possible to consider interactions with other avatars or objects, thus creating a sort of awe-inspiring virtual world (e.g., Bartle, 2004; Triberti and Chirico, 2016). Further, it would be useful to consider whether the component of interaction acts differently in a natural context, compared to a virtual one. Moreover, VR provides the possibility to design almost infinite scenarios including different objects and characters users can interact with. This allows for planning tasks and challenges within the VE. Further, VR allows for a complete tracking of participants’ performance. As previously mentioned, another advantage provided by VR compared to 360° videos is that VE can reproduce “paradoxical” phenomena (i.e., violating laws of physics) in controlled settings. This could be one of the assets that can contribute to the natural ability of VR to induce a need for accommodation. Regarding the sense of physical presence, we considered effect size from the comparison between 360° awe vs. 360° neutral environment (Chirico et al., 2017) with those from this study (Forest vs. Neutral; Mountain vs. Neutral; Earth view vs. Neutral). Indeed, results indicated a higher effect size in the 360° study. However, in any case, effect size was based on the comparison with a neutral condition. It could be possible that this VR format could enhance the sense of physical presence even for neutral stimuli, as a matter of medium. Thus, the differences between emotional VEs and Neutral one were lower.

Finally, at the level of usability, these environments have been highly tolerated by participants.

In short, these validated environments can be used in different contexts and propose some design guidelines to be followed when creating an awe-inspiring VE.

For instance, it is possible to design multiple and continuative experiences in VR such as a VR training for repeated awe induction, maybe in combination with conventional emotion induction technique, such as emotional recall (e.g., Gilet, 2008; Gaggioli et al., 2014). This repeated but controlled exposure could lead to longer-term positive outcomes for individuals’ wellbeing and health. With this regard, it is possible to imagine several applications for awe, related to its positive consequences for health and wellbeing. For instance, it would be possible to integrate a biofeedback device with one of these validated VEs helping participants recognizing and self-inducing and regulating intense awe emotional states in an ecological way or sharing feelings of awe with another person at the physiological level (e.g., Neidlinger et al., 2017). All people could navigate in these environments and benefit from their effects. It is possible to design different training to empower several cognitive, social or emotional skills. Indeed, awe can enhance our prosocial attitude toward even unknown others (Piff et al., 2015), it can improve our ability to manage stress (Stellar et al., 2015), or it can increase the satisfaction toward our life. Interestingly, awe can affect even the perception of our body, thus making feel people smaller than they actually are. All these aspects can be potential targets of an awe-inducing training.

We are on the edge of a new modality to design complex emotions fully exploiting their unique potential for human progress and wellbeing.

### Limitations

This is the first study testing the potential of VR in inducing awe. As a preliminary and explorative study, some limitations exist. First, we used the conventional single item to assess awe but more sophisticated instruments of assessment could be considered to measure this complex emotions, such as psychophysiological measures, as it has been already successfully done (Chirico et al., 2017). In this specific case, an eye-tracking system could be used to track participants’ fixations points and saccades, to determine environmental cues participants focused on more. This would help design effective awe-inducing environments. More, this is an exploratory study in which the aim was to test the potential of immersive and interactive VR in inducing awe. However, it could be possible to test whether immersive and interactive VR is more effective than simply immersive VR systems in inducing this complex emotion. More, we assessed only seven other potentially intervenient emotions besides awe, but future works should consider also other positive and negative emotions that could co-occur with awe (for instance, see Hofmann et al., 2017). Finally, we did not consider the role of individuals’ proneness to live discrete positive emotions, in the likelihood to experience awe in response to these environments. Personality and stable dispositional factors resulted as relevant for other positive emotions such as amusement (Pietquin and Dupont, 2011). Therefore, they should be considered in future studies to define a more comprehensive model of awe and self-transcendent experiences in VR.

## Author Contributions

Authors contributed according to their competences and interests. AC and AG conceived the main idea of the article. AC collected all data and carried out statistical analyses. LC and FF conceived and developed the technical setup. AC wrote the first draft of the manuscript, while AG, FF, and LC contributed to the final writing of the manuscript by giving suggestions regarding the issues related to the rhetoric and to the literature. AG supervised the entire work. All authors contributed to the manuscript, read, and approved the final version.

## Conflict of Interest Statement

The authors declare that the research was conducted in the absence of any commercial or financial relationships that could be construed as a potential conflict of interest.
